# Antioxidant, Anti-Inflammatory and Antiproliferative Effects of the *Vitis vinifera* L. var. Fetească Neagră and Pinot Noir Pomace Extracts

**DOI:** 10.3389/fphar.2020.00990

**Published:** 2020-07-03

**Authors:** Ştefania Silvia Balea, Alina Elena Pârvu, Marcel Pârvu, Laurian Vlase, Cristina Adriana Dehelean, Tiberia Ioana Pop

**Affiliations:** ^1^ Department of Horticulture and Landscaping, Faculty of Horticulture, University of Agricultural Sciences and Veterinary Medicine, Cluj-Napoca, Romania; ^2^ Department of Pathophysiology, Faculty of Medicine, “Iuliu Hatieganu” University of Medicine and Pharmacy, Cluj-Napoca, Romania; ^3^ Department of Biology, Faculty of Biology and Geology, Babes-Bolyai University, Cluj-Napoca, Romania; ^4^ Department of Pharmaceutical Technology and Biopharmaceutics, Faculty of Pharmacy, “Iuliu Hatieganu” University of Medicine and Pharmacy, Cluj-Napoca, Romania; ^5^ Department of Toxicology and Drug Industry, Faculty of Pharmacy, “Victor Babeș” University of Medicine and Pharmacy, Timișoara, Romania; ^6^ Department of Technical and Soil Sciences, Faculty of Agriculture, University of Agricultural Sciences and Veterinary Medicine, Cluj-Napoca, Romania

**Keywords:** grape pomace, Fetească neagră, Pinot noir, antioxidant, anti-inflammatory, antiproliferative

## Abstract

The pathophysiology of inflammation and oxidative stress generated during different types of cancers and anticancer treatments is well documented. Traditionally, grape pomace is used for animal feed, organic fertilizers, ethanol production or is disposed as waste. Because grape pomace is a rich source of antioxidant compounds, the purpose of the study was to evaluate the antioxidant, anti-inflammatory, and antiproliferative effects of fresh and fermented grape pomace extracts of two *Vitis vinifera* L. varieties Fetească neagră and Pinot noir cultivated in Romania. Firstly, grape pomace phytochemical analysis and *in vitro* antioxidant tests were performed. Secondly, the effect of a seven-day pretreatment with grape pomace extracts on the turpentine oil-induced inflammation in rats was assessed by measuring total oxidative status, total antioxidant response, oxidative stress index, malondialdehyde, total thiols, nitric oxide and 3-nitrotyrosine. Thirdly, the antiproliferative properties were evaluated on human lung carcinoma (A549), human breast adenocarcinoma (MDA-MB-231), murine melanoma (B164A5), and keratinocyte (HaCat) cell lines. Fetească neagră and Pinot noir grape pomace extracts have a rich content of polyphenols and *in vitro* antioxidant effect. Fermented samples had higher polyphenol content, but fresh samples had better antioxidant activity. Pretreatment with grape pomace extracts reduced inflammation-induced oxidative stress in a concentration-dependent way, fresh samples being more efficient. The malignant cells’ proliferation was inhibited by all grape pomace extracts, fermented Fetească neagră extracts having the strongest effect. Conclusion: fresh and fermented pomace extracts of *Vitis vinifera* L. varieties Fetească neagră and Pinot noir cultivated in a Romanian wine region have antioxidant, anti-inflammatory and antiproliferative effects.

## Introduction

During tumor development tissue hypoxia occurs, which activates signaling pathways stimulating cell proliferation, angiogenesis, and death. Hypoxia in the tumor microenvironment causes tumor cells to secrete chemokines, such as interleukin-1 (IL-1), tumor necrosis factor alpha (TNF-alpha), or interleukin 8 (IL-8), which activates neutrophils to produce more pro-oncogenetic and immunosuppressive factors (Ardi et al., 2007). Lymphocytes are an important part of the immune response to tumors because they inhibit tumorigenesis and kill cancer cells. However, in a proinflammatory tumor state, neutrophils can suppress the efficient lymphocyte-mediated immune response. Moreover, there are tumor-derived factors that induce myelopoiesis, accumulation, and differentiation of tumor-associated macrophages (TAMs). These TAMs produce ROS and RNS in the tumor microenvironment, triggering a tumor-induced inflammation, and creating a vicious cycle between inflammation and cancer (Ardi et al., 2007; Reuter et al., 2010; Andrisic et al., 2018; de Souza et al., 2018). As for platelets, thrombocytosis is common in cancer because tumor cells secrete thrombopoietic cytokines, such as interleukin-6 (IL-6). In turn, platelets can promote angiogenesis through vascular endothelial growth factor (VEGF) secretion and thus protect tumor cells from the immune response. Cancer cells’ adaption to hypoxia is part of the malignant phenotype and aggressive tumor progression mechanism (Bambace and Holmes, 2011; Petrillo et al., 2018).

Another issue related to the tumor-induced inflammation is that it modulates cancer responsiveness or resistance to anticancer therapies. In some cancers, elevated basal nuclear transcription factor NF-*κ*B activity and inflammatory mediator production were associated with tumor resistance to chemotherapy and radiation. Chemotherapy with cisplatin, daunomycin, doxorubicin, 5-fluorouracil, paclitaxel, tamoxifen, vinblastine, and vincristine may cause chemoresistance by activating the NF-*κ*B, and NF-*κ*B inhibition acts as radiosensitizer of the tumor cells (Silva et al., 2018). Because oxygen is the best radiosensitizer, tumor-induced hypoxia is considered to be the most important cause of radioresistance (Reuter et al., 2010; Kim et al., 2018).

In order to interrupt the vicious circle between inflammation, nitro-oxidative stress, and cancer, the endogenous enzymatic and nonenzymatic antioxidant molecules may be supplemented with exogenous antioxidant molecules, such as plant-derived polyphenolic compounds. In high concentrations, high pH, and the presence of redox-active metals, polyphenolic compounds can exert a pro-oxidant effect with cytotoxic consequences (Pizzino et al., 2017).

Grape pomace (GP) is a residue of the winemaking process and represents an important ecological and economic problem of waste management, since around 20% of the grapes weight remains as GP (Beres et al., 2017). Due to the incomplete extraction during the winemaking process, around 70% of polyphenolic compounds remain in fermented GP. Traditionally it is mainly used for animal feed, organic fertilizers, ethanol production or is disposed as waste. Over the last decades many products were obtained from grape pomace, and the most common approach was to prepare GP extracts (García-Lomillo and González-SanJosé, 2017). In some countries GP is included in functional foods and cosmetic preparations. Due to its polyphenol content with strong antioxidant effect, GP seemed to be efficient for the prevention of disease-associated oxidative stress. Other GP effects are the antimicrobial and anti-inflammatory activities (Teixeira et al., 2014).

The aims of the study were to evaluate the antioxidant, anti-inflammatory, and antiproliferative effects of the fresh and fermented GP ethanol extracts from two *Vitis vinifera* L. varieties, Fetească neagră and Pinot noir, cultivated in Romania.

## Materials and Methods

### Reagents and Cell Cultures

Sulfanylamide (SULF), N-(1-Naphthyl) ethylenediamine dihydrochloric acid (NEDD), vanadium chloride (III) (VCl3), methanol, diethyl ether, xylenol orange [o-cresosulfonphthalein-3,3-bis (sodium methyliminodiacetate)], orthodianisidine dihydrochloric acid (3-3′-dimethoxybenzidine), ferrous ammonium sulfate, hydrogen peroxide (H2O2), sulfuric acid, hydrochloric acid, glycerol, trichloroacetic acid (TCA), ethylenediaminetetra-acetic acid, sodium dodecal, sulfate butylated hydroxytoluene, thiobarbituric acid, 1,1,3,3-tetraethoxypropane, 2,4-dinitrophenylhydrazine (DNPH), 5,5′-dithionitrobis 2-nitrobenzoic acid (DTNB), 1,1-diphenyl-2-picrylhydrazyl (DPPH), o-phthalaldehyde (Darmstadt, Germany) were purchased from Merck and Sigma-Aldrich (Taufkirchen, Germany), 96% ethanol (SA, Iași, Romania), ascorbic acid (Lach-Ner, Czech Republic). All chemicals were of analysis grade.

A549—human lung carcinoma, MDA-MB-231—human breast adenocarcinoma, B164A5—murine melanoma cell lines, and HaCat keratinocyte cell lines were purchased from the European Cell Culture Collection (ECACC). Dulbecco’s Modified Eagle Environment (DMEM), fetal calf serum (FCS) and resazurine sodium salt were purchased from Sigma Aldrich (Munich, Germany). The phosphate buffer solution (PBS) and the mixture of penicillin/streptomycin and trypsin-EDTA antibiotics were purchased from Gibco (Karlsruhe, Germany).

The ELISA kit for 3-nitrotyrosine (3NT) (KA0445) was purchased from ABNOVA EMBLEM (Heidelberg, Germany).

### Grape Samples

The *Vitis vinifera* L. variety Fetească neagră (clone 762 grafted on rootstock SO4, Austria), and *Vitis vinifera* L. variety Pinot noir (clone 828 grafted on rootstock SO4, France) planted in 2006, in Mureș County, Mica parish, part of Târnavelor Plateau (46°21′44.5″N and 24°23′55.7″E; 330–350 m above sea level), Romania, were used in our study. Grapes were harvested manually at full maturity level during the 2018 vintage. The GP samples were collected in two winemaking stages: the fresh unfermented GP was supplied immediately after pressing the grapes, and the fermented GP was supplied after 20 days of fermentation at 20°C and must separation. The samples were stored in vacuum bags at −22°C prior to the analysis and use in the experiments.

### Plant Extract Preparation

Fetească neagră and Pinot noir fresh and fermented GP extracts were obtained with 70% ethanol (Merck, București, Romania) by a modified Squibb repercolation method (1/1 g/ml) (Andreicut et al., 2018).

### Determination of Total Polyphenols Content

The total polyphenol content (TPC) of the extracts was measured using the Folin–Ciocâlteu method, with some modifications. The absorbance was measured at 760 nm using a JASCO UV-VIS spectrophotometer. Standard curve was prepared using different concentrations of gallic acid (GAE). TPC was expressed as mg GAE/g dry plant material (Toiu et al., 2018).

### LC/MS Analysis of Polyphenolic Compounds

A HPLC-MS method was used for the qualitative and quantitative polyphenol determination. The analysis was carried out with an Agilent 1100 Series HPLC system (Agilent, USA) consisting of a G1322A degasser, G1311A binary gradient pump and a G1313A autosampler, and a UV detector. The chromatographic separation was performed using a reversed-phase analytical column (Zorbax SB-C18 100 mm × 3.0 mm i.d., 3.5 µm particle) maintained at 48°C. The mobile phase consisted of a binary gradient: methanol and acetic acid 0.1% (v/v). The mobile phase was delivered with a flow rate of 1 ml/min and the injection volume was 5 µl. Polyphenol detection was performed on UV (330 and 370 nm) and MS mode. The MS system operated using an ion trap mass spectrometer with electrospray negative ionization. The chromatographic data were processed using Chem station and Data Analysis software from Agilent, USA. The calibration curves in the 0.5–5 µg/ml range showed good linearity (R2 < 0.999) for a five point plot (Toiu et al., 2018; Farcaș et al., 2019).

For the LC/MS profile polyphenolic compounds standards were: caftaric acid, hyperoside, isoquercitrin, rutoside, miricetol, quercitrin, quercetol, and kaempferol.

### *In Vitro* Antioxidant Activity Analysis

The antioxidant activity (AOA) of the extracts was evaluated by DPPH radical scavenging assay (Blois, 1958). DPPH is considered a stable radical because of the paramagnetism conferred by its odd electron. DPPH solution in ethanol 96% with a concentration of 1 mM was used as a standard antioxidant stock solution. In each reaction, 0.5 ml of the GP extracts was mixed with 0.5 ml of 1 mM DPPH and with 2 ml of 0.167 mM ascorbic acid in ethanol 96%. The mixture was analyzed using a UVI Line 9400 spectrophotometer (SI Analytics), for 20 min at 10 s intervals. Ascorbic acid was used as positive control. The reduction of DPPH free radicals was measured by reading the absorbance at 516 nm. DPPH is a purple colored stable free radical and when reduced, it becomes yellow. The AOA-percentage was calculated with the following formula:

$$\text{AOA}(\%)=100-\frac{\text{A}\;\mathrm{sample}}{\text{A}\;\mathrm{control}}\times 100$$

where: AOA = antioxidant activity (%); A *control* = absorbance of DPPH measured at 516 nm, for 20 min at an interval of 10 s (without sample); A *sample* = absorbance of the sample measured at 516 nm, for 20 min at an interval of 10 s.

### Animals and Experimental Protocol

The experiments were performed in triplicate on 14 groups (n = 5) of male albino Wistar rats, weighing 200–250 g that were bred in the Animal Facility of the Iuliu Hațieganu University of Medicine and Pharmacy, Cluj-Napoca, Romania. The animals were housed in standard polypropylene cages (five per cage) under controlled conditions (12 h light/dark cycle at an average temperature of 21–22°C) and with *ad libitum* access to standard pellet diet (Cantacuzino Institute, Bucharest, Romania) and water. Experimental protocols have been approved by the Ethics Committee (nr. 26/16.12.2015) of the Faculty of Veterinary Medicine, University of Agricultural Sciences and Veterinary Medicine, Cluj-Napoca, Romania. Four ethanolic extracts of GP were tested: FNfr—Fetească neagră fresh GP extract; FNfe—Fetească neagră fermented GP extract; PNfr—Pinot noir fresh GP extract; PNfe—Pinot noir fermented GP extract. The extracts were administered orally by gavage (1 ml/animal/day) in three dilutions, respectively 100, 50, and 25%, for seven days. Animals from the negative control group (CONTROL) and the inflammation group (INFLAM) received tap water (1 ml/animal/day) by gavage for seven days (He and Mu, 2015). On day eight, except for the CONTROL group, inflammation was induced by injecting turpentine oil (6 ml/kg b.w.) intramuscularly (Toyohara et al., 2013). On day nine, under general anesthesia induced by pentobarbital (50 mg/kg IP) (Zatroch et al., 2017), blood was withdrawn by retro-orbital puncture, serum was separated and stored at −80°C until use, and animals were euthanized by cervical dislocation.

### *In Vivo* Oxidative Stress Assessment

Oxidative stress was assessed using global and specific tests. The global oxidative stress tests were total oxidative status (TOS), total antioxidant reactivity (TAR), and the oxidative stress index (OSI). Specific oxidative stress tests were malondialdehyde (MDA), total thiols (SH), total serum nitrates and nitrates (NOx), and 3-nitrotyrosine (3NT) (Balea et al., 2018b; Farcas et al., 2019).

### Alamar Blue Cell Proliferation Assay

The four cell lines were seeded on 96-well microplates (1 × 104 cells/well). After 24 h incubation (37°C, 5%CO_2_ and 95% humidity), 200 µl of medium containing DMEM supplemented with 10% FCS, 1% mixture of penicillin/streptomycin (100 U/ml penicillin and 100 pg/ml streptomycin) and ethanolic extracts of GP (1,000 µg/L) were added to each well and incubated for 48 h (Lo et al., 2011). After 48 h incubation, 20 µl of Alamar blue (AB) was added to each well, and cells were incubated for 4 h at 37°C. AB staining was used to determine the cell viability of both cancer cells (A549, MDA-MB-231, B164A5) and healthy HaCat cells after they were stimulated with GP extracts. The plate was then placed under a microplate reader to determine the absorbance value of each well at 570 and 600 nm; untreated cell wells were used as controls. All *in vitro* experiments were performed in triplicate (Sadej and Skladanowski, 2012). Cell proliferation was calculated by the formula:

$$\text{\% AB reduction}=\frac{\left(\varepsilon \text{OX}\right)\lambda 2\left(\text{A}\lambda 1\right)-\left(\varepsilon \text{OX}\right)\lambda 1\left(\text{A}\lambda 2\right)}{\left(\varepsilon \text{RED}\right)\lambda 1\left(\text{A}{}^{\circ}\lambda 2\right)-\left(\varepsilon \text{RED}\right)\lambda 2\left(\text{A}{}^{\circ}\lambda 1\right)}\times 100$$

where: εOX = the molar extinction coefficient of the oxidized AB form (blue), A = absorption of test wells, A° = absorption of the positive growth control well (cells without tested compounds), λ1 = 570 nm, λ2 = 600 nm.

### Statistical Analysis

All results were expressed as mean ± standard deviation (SD) whenever data were normally distributed. Comparisons between the different experimental groups were performed using the ANOVA test and the *post hoc* Bonferroni–Holm test. The correlation analysis was performed with the Pearson test. Values of p < 0.05 were considered statistically significant. The analysis was performed using IBM SPSS Statistics, version 20 (SPSS Inc. Chicago, IL, USA).

## Results and Discussion

Cancer remains a leading cause of death worldwide despite considerable progress in basic research and clinical studies. Early diagnosis and chemoprevention are essential for reducing the incidence of cancers. In addition, the side effects of conventional therapies contribute to diminishing patients’ life quality and imply the need to develop a safe and effective therapeutic alternative. Although research has been conducted to combat cancer in terms of natural therapy, a satisfactory and complete therapeutic agent has not been found.

### Polyphenols Analysis

The differences between the polyphenol content depend on the grape variety, grape maturity, environmental factors, and the technological processes used during the vinification (Xu et al., 2016). The TPC of the extracts varied with the GP product, FNfe having the higher TPC (15.03 ± 0.84 mg GAE/g), followed by PNfe (9.23 ± 0.85 mg GAE/g), PNfr (8 ± 0.10 mg GAE/g), and FNfr (6 ± 0.75 mg GAE/g).

The LC/MS analysis identified the compounds from the GP extracts and confirmed the TPC results, respectively fermented GP samples had a higher content of polyphenols than the fresh GP samples, FNfe having the highest concentration of polyphenols.

**Figure 1:**
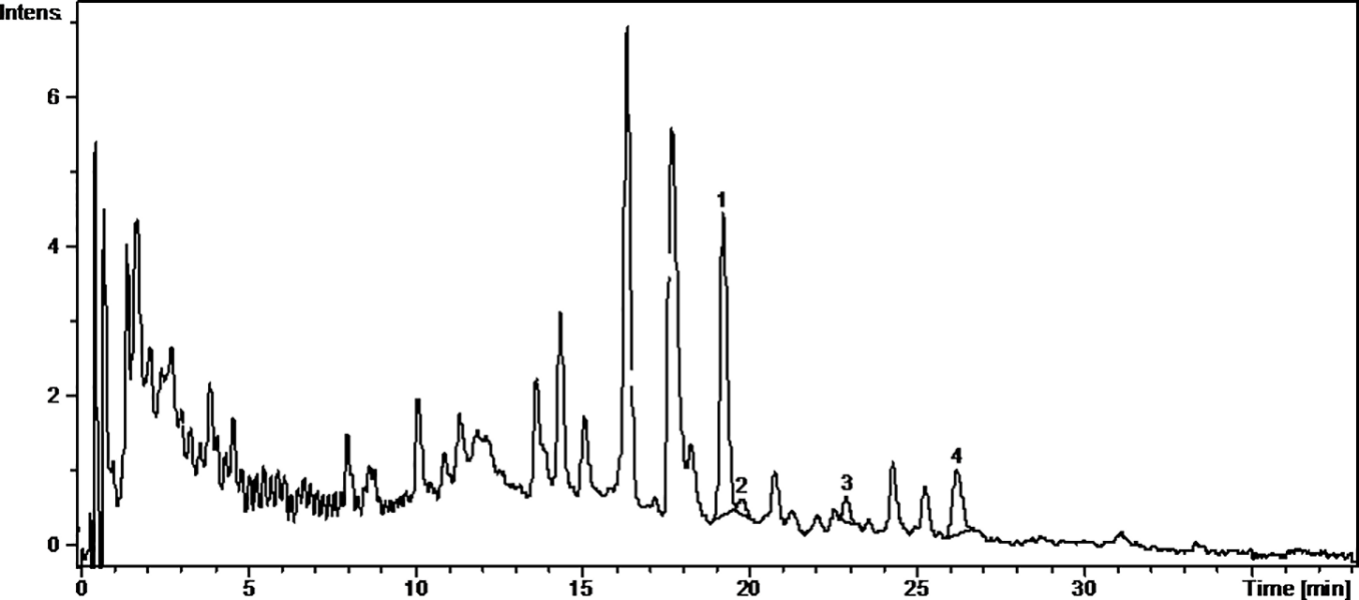
The UV chromatogram of fresh Pinot noir 2015 grape pomace extract. 1, Isoquercitrin; 2, Rutoside; 3, Quercitrin; 4, Quercetin.

Caftaric acid is a phenolic acid found in grapes and gives the white wine color (Song et al., 2018a). In this study, caftaric acid was below the limit of detection (LOD) for all analyzed samples (**Table 1**, **Figures 1** **–** **4**).

**Table 1:** The: polyphenolic compounds content in the Fetească neagră and Pinot noir grape pomace extracts.

Compound (µg/ml)	FNfr	FNfe	PNfr	PNfe
**Caftaric acid**	NF	NF	NF	NF
**Kaempferol**	3.679 ± 0.04	5.740 ± 0.78	NF	NF
**Miricetol**	0.341 ± 0.01	1.029 ± 0.12	NF	NF
**Isoquercitrin**	2.429 ± 0.18	65.698 ± 7.11	3.685 ± 0.35	42.042 ± 1.35
**Hyperoside**	0.804 ± 0.06	10.813 ± 0.18	NF	NF
**Rutoside**	NF	NF	NF	2.136 ± 0.21
**Quercitrin**	NF	14.952 ± 1.54	NF	3.272 ± 0.169
**Quercetol**	8.407 ± 0.54	15.637 ± 1.18	2.473 ± 0.22	3.936 ± 0.27

FNfe, Fetească neagră fermented grape pomace extract; FNfr, Fetească neagră fresh grape pomace extract; PNfe, Pinot noir fermented grape pomace extract; PNfr, Pinot noir fresh grape pomace extract; NF, not found, below the Limit of Detection. Values are expressed as mean ± SD (n = 3).

**Figure 2:**
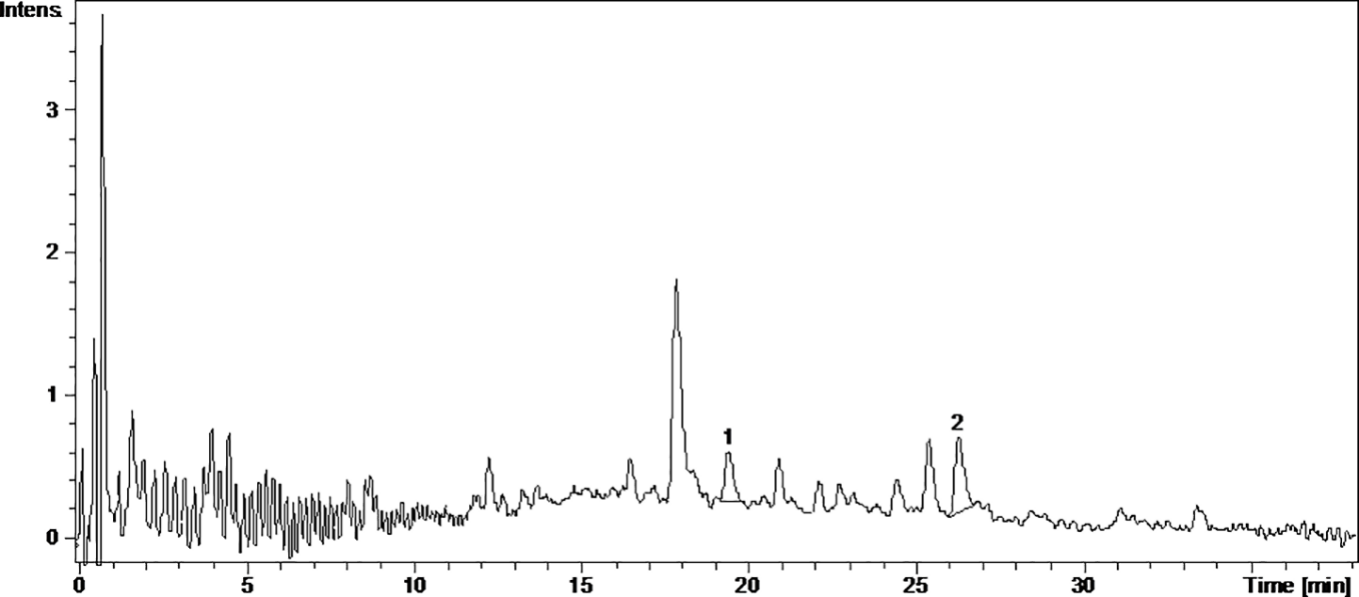
The UV chromatogram of frtmented Pinot noir 2015 grape pomace extract. 1, Isoquercitrin; 2, Quercetin.

**Figure 3:**
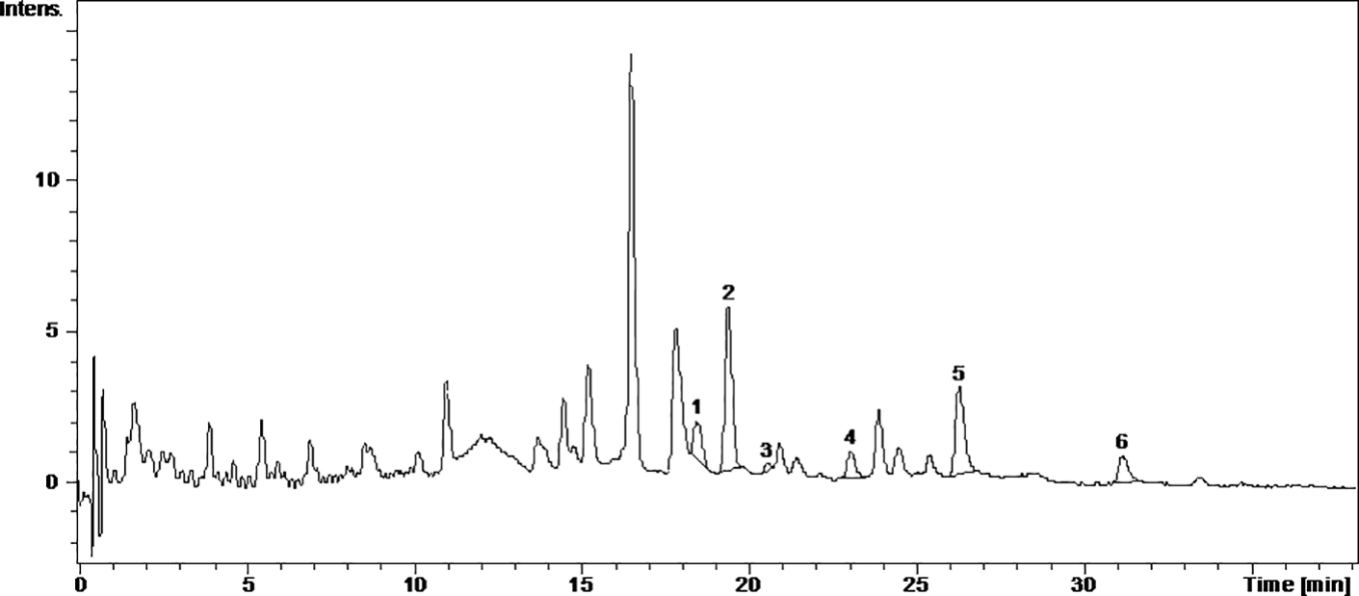
The UV chromatogram of fresh Fetească neagră 2015 grape pomace extract. 1, Hyperoside; 2, Isoquercitrin; 3, Miricetol; 4, Quercitrin; 5, Quercetin; 6, Kaempferol.

**Figure 4:**
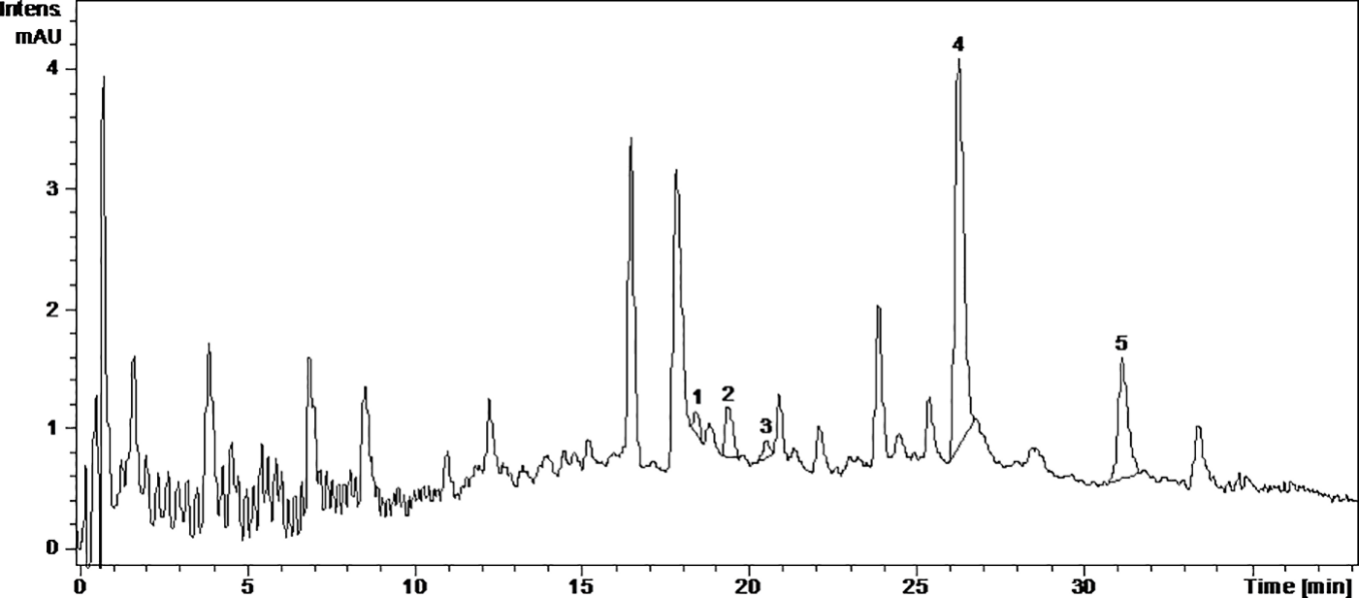
The UV chromatogram of fermented Fetească neagră 2015 grape pomace extract. 1, Hyperoside; 2, Isoquercitrin; 3, Miricetol; 4, Quercetin; 5, Kaempferol.

Kaempferol is a dietary antioxidant flavonol that reduces the risk of chronic diseases, including cancer. At the molecular level, kaempferol has been reported to modulate a number of key elements in cell signal transduction related to apoptosis, angiogenesis, inflammation, and metastasis (Chen and Chen, 2014; Beres et al., 2017). It was found that exposure to solar radiation increase kaempferol concentration. It was detected only in the FNfr (3.679 ± 0.04 µg/ml) and Fnfe (5.740 ± 0.78 µg/ml) GP extracts, and the concentration was comparable to the one determined in South African Shiraz (0.36 mg/100 ml) and Cabernet Sauvignon (0.35 mg/100 ml), but higher than the concentration from three Calabrian red wines (Gidaro et al., 2016) (**Table 1**, **Figures 1** **–** **4**).

Miricetol is a flavonol with potent antioxidant, anticancer, analgesic, antidiabetic, hepatoprotective and anti-inflammatory activities. Extensive research into the anticancer activities of miricetol has shown that the compound is cytotoxic to a number of human cancer cell lines, including liver, skin, colon, and pancreas cancer cells. The antioxidant property of miricetol was higher than that of vitamin E. The anti-inflammatory activity of miricetol has been demonstrated in acute and chronic *in vivo* animal models by preventing NF-kB activation, NO, proinflammatory cytokines and PGE2 production (Semwal et al., 2016). In the Pinot noir GP extracts, kaempferol and miricetol were under the LOD, in FNfe moderate amounts of kaempferol and small amounts of miricetol were detected, and in FNfr only small amounts of miricetol were found (**Table 1**, **Figures 1** **–** **4**).

Quercetin exists mostly in its quercetin glycosides, which occur naturally and are among the most common flavonoids in the human diet. They have neuroprotective, cardioprotective, chemopreventive, antioxidant, anti-inflammatory, and antiallergic effects. The antioxidant and anti-inflammatory effects were associated with reduced expression of iNOS and inhibition of NF-*κ*B expression (Dai et al., 2013). Quercetin has poor bioavailability, but quercetin glycosides have the same *in vivo* therapeutic effects and better bioavailability. The quercetin glycosides evaluated in our study were quercitrin, isoquercetin, hyperoside, and rutoside (Song et al., 2018b). Quercitrin was above the LOD only in the fermented GP samples, FNfe having a significantly higher concentration of quercitrin than PNfe (p < 0.001). Isoquercetin was present in all the samples, having a significantly higher concentration in FNfe and PNfe (p < 0.001). Hyperoside was found above the LOD only in Fetească neagră samples, FNfe having a more important content of hyperoside than FNfr (p < 0.001). Rutoside has been shown to have immunomodulatory (Ganeshpurkar and Saluja, 2017), antioxidant (Shahid et al., 2016), anti-inflammatory, neuroprotective (Song et al., 2018b), antitumor (Chen et al., 2015), and cardioprotection (Wang et al., 2017) effects. As flavonol, rutoside has low bioavailability due to poor absorption, high metabolism, and rapid excretion, which limits its potential therapeutic use (Martinez-zapata et al., 2016). Rutoside was detected only in the PNfe samples (**Table 1**, **Figures 1** **–** **4**).

Quercetol has antioxidant properties by inhibing lipid peroxidation and xanthine oxidase, by scavenging ROS *in vitro* and can inhibit cancer (Ali et al., 2016). Quercetol was found in all GP samples, FNfe having the higher concentration (**Table 1**, **Figures 1** **–** **4**).

In a previous study we found significant concentrations of resveratrol in the Fetească neagră and Pinot noir GP extracts, the fresh GP extracts having a higher concentration (Balea et al., 2018a; Balea et al., 2018b). Several *in vitro* studies have shown that resveratrol has antitumor, antioxidant, anti-inflammatory, cardioprotective and antiplatelet activity, and glycosylated stilbenes have antifungal and antioxidant effects (Flamini et al., 2013). Studies conducted with resveratrol showed that it improves the effectiveness of cisplatin and doxorubicin chemotherapy, suggesting that it can be used in cervical cancer treatment (Silva et al., 2018).

In conclusion, the polyphenol analysis of the fresh and fermented pomace of *Vitis vinifera* L. var. Fetească neagră and var. Pinot noir extracts performed in the present and previous studies (Balea et al., 2018b) suggested antioxidant, anti-inflammatory and antiproliferative activities.

### *In Vitro* Antioxidant Activity

**Figure 5:**
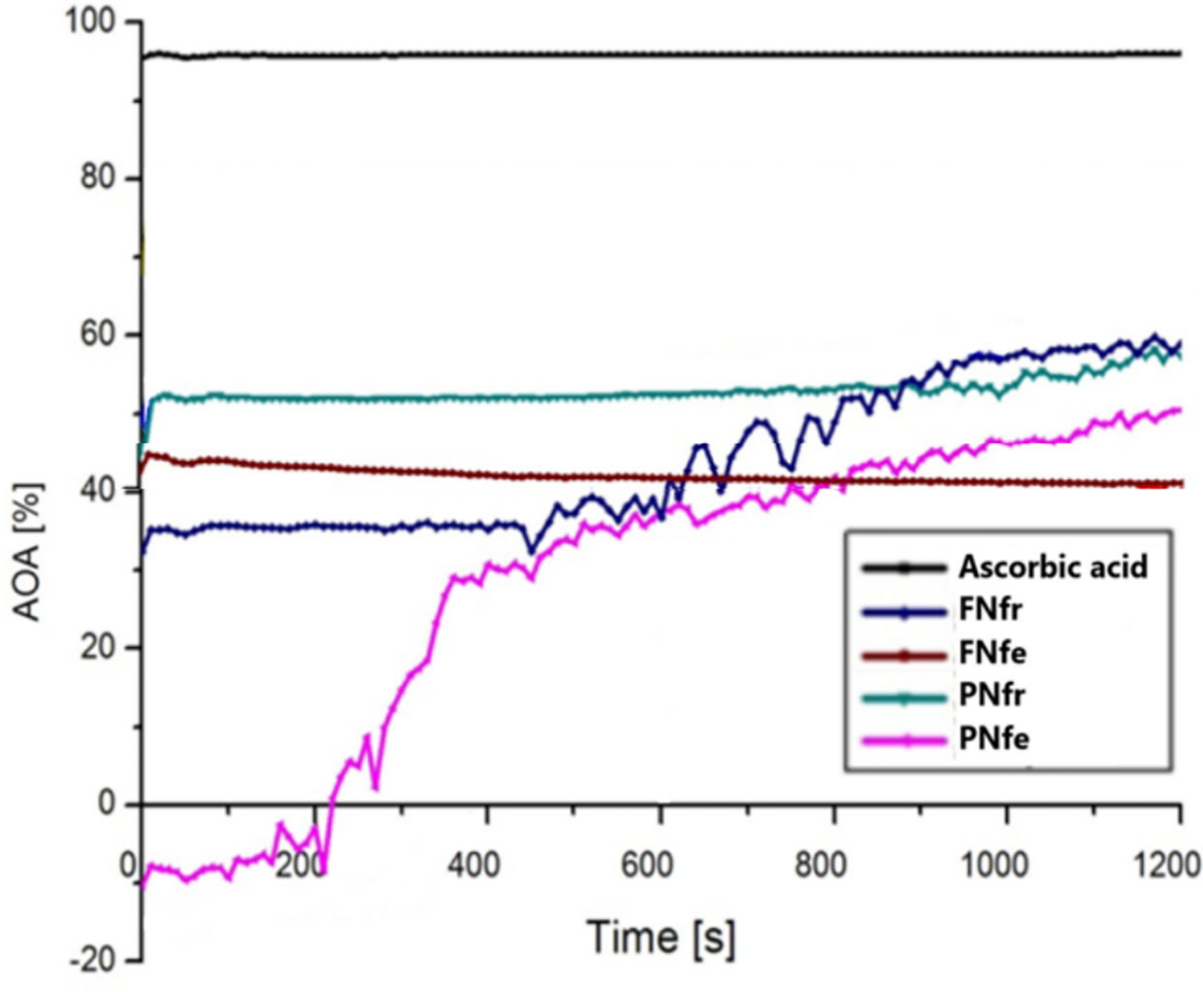
Antioxidant activity evaluation with DPPH test. FNfe, Fetească neagră fermented grape pomace extract; FNfr, Fetească neagră fresh grape pomace extract; PNfe, Pinot noir fermented grape pomace extract; PNfr, Pinot noir fresh grape pomace extract.

All ethanolic extracts of GP proved to have lower antioxidant effects compared to the ascorbic acid. The fresh extracts had a higher AOA after 1,200 s than in the initial moment, FNfr increasing AOA to around 65%, and PNfr increasing AOA to around 60%. The PNfe extracts initially had no antioxidant effects, but after 1,200 s AOA increased to 50%. The FNfe was the single sample that revealed a slight decrease of the AOA after 1,200 s, from around 44% initially to 40%. The AOA results do not correlate with the polyphenol content because the fresh GP samples had a higher AOA and the fermented GP samples had a higher polyphenol concentration (**Figure 5**).

### *In Vivo* Antioxidant Effects

Polyphenols can reduce oxidative stress directly by preventing free radical formation and indirectly by increasing the activity of key antioxidant enzymes (Annunziata et al., 2020). While some studies have shown that natural extracts have antioxidant properties both *in vitro* and *in vivo*, other studies have shown that *in vitro* antioxidant activity does not always apply to *in vivo* models because polyphenols may also act as prooxidants (Veskoukis et al., 2012). Therefore, after demonstrating the antioxidant capacity by DPPH test, the *in vivo* antioxidant activity was tested on a turpentine-induced acute inflammation model (Lim et al., 2013). Because ROS have a short life, the *in vivo* oxidative stress assessment is generally based on the measurement of indirect markers. There are global parameters, like TOS, TAR, OSI, and NOx, and specific tests, such as molecules modified by free radicals, antioxidant enzymes, and transcription factors (Alam et al., 2013).

Turpentine administration resulted in a significant increase in TOS and OSI (p < 0.001) plus a TAR reduction (p < 0.001). Overall, the global oxidative stress parameters revealed important antioxidant properties for the evaluated GP extracts. The GP extract effects on TOS and OSI were concentration-dependent and decreased significantly TOS and OSI (p < 0.001), but there were no important changes of TAR (p > 0.05). Related to TOS and OSI, between the two *Vitis vinifera* L. varieties or between fresh and fermented GP extracts, there were no significant differences. The fermented GP samples had higher polyphenol content but that did not correlate with TOS and OSI changes (**Table 2**).

**Table 2:** *In vivo* antioxidant global tests results.

GROUP	OSI	TOS (µM H_2_O_2_/L)	TAR (mM TROLOX/L)
**CONTROL**	0.25 ± 0.02	26.92 ± 10.30	1.09 ± 0.0005
**INF**	0.37 ± 0.03	40.51 ± 3.34	1.09 ± 0.0008
**FNfe 100%**	0.26 ± 0.05	28.06 ± 5.39	1.09 ± 0.0009
**FNfe 50%**	0.21 ± 0.03	23.38 ± 2.80	1.09 ± 0.0007
**FNfe 25%**	0.27 ± 0.08	29.35 ± 8.25	1.09 ± 0.0001
**FNfr 100%**	0.26 ± 0.04	28.84 ± 4.44	1.09 ± 0.0005
**FNfr 50%**	0.24 ± 0.04	25.72 ± 4.64	1.09 ± 0.0006
**FNfr 25%**	0.29 ± 0.08	31.41 ± 8.25	1.09 ± 0.0012
**PNfr 100%**	0.26 ± 0.03	28.08 ± 3.39	1.09 ± 0.0004
**PNfr 50%**	0.27 ± 0.03	29.41 ± 2.87	1.09 ± 0.0006
**PNfr 25%**	0.28 ± 0.02	30.16 ± 1.97	1.09 ± 0.0003
**PNfe 100%**	0.26 ± 0.02	28.05 ± 2.82	1.09 ± 0.0010
**PNfe 50%**	0.28 ± 0.05	30.21 ± 4.93	1.09 ± 0.0010
**PNfe 25%**	0.29 ± 0.06	31.31 ± 7.01	1.09 ± 0.0007

FNfe, Fetească neagră fermented grape pomace extract; FNfr, Fetească neagră fresh grape pomace extract; PNfe, Pinot noir fermented grape pomace extract; PNfr, Pinot noir fresh grape pomace extract; TOS, total oxidative status; TAR, total antioxidant reactivity; OSI, oxidative stress index.

An important cellular effect of ROS is peroxidation of the phospholipids and fatty acids in the membrane, resulting in modified membrane fluidity, protein structure, and cell signaling. Such a lipid peroxidation product is MDA. Some studies indicated that MDA has mutagenic and tumor promoter potential. Therefore, we evaluated the effects of GP extracts on the MDA formation induced by the experimental inflammation. Turpentine administration increased MDA significantly (p < 0.01). All GP extracts had moderate inhibitory effect on MDA formation (p < 0.01). These results may be linked to the finding that flavan-3-ols monomers reduce LDL oxidizability through their incorporation into the LDL particles and the radical trapping effects (Annunziata et al., 2018; Annunziata et al., 2019) and to our previous study which reported an important content of flavan-3-ols monomers in the GP extracts (Balea et al., 2018b; Balea et al., 2018a). There were no significant differences between the effects on MDA of the two grape varieties or between fresh and fermented GP extracts of the same variety (p > 0.05) (**Table 3**).

**Table 3:** *In vivo* antioxidant specific tests results.

GROUP	MDA (nM/L)	SH (mM GSH/L)	NO (µM/L)	3NT (nmol/L)
**CONTROL**	1.09 ± 0.22	0.54 ± 0.06	33.31 ± 4.50	29.78 ± 2.39
**INF**	4.46 ± 0.50	0.47 ± 0.11	40.04 ± 5.44	34.76 ± 6.32
**FNfe 100%**	4.15 ± 0.71	0.68 ± 0.18	28.97 ± 1.76	37.80 ± 3.14
**FNfe 50%**	4.45 ± 0.97	0.47 ± 0.14	36.59 ± 3.79	41.80 ± 6.48
**FNfe 25%**	3.95 ± 0.65	0.59 ± 0.16	39.77 ± 6.29	50.40 ± 7.42
**FNfr 100%**	3.84 ± 0.62	0.58 ± 0.09	44.07 ± 6.20	30.70 ± 2.42
**FNfr 50%**	3.74 ± 0.40	0.43 ± 0.10	47.31 ± 2.41	40.50 ± 5.01
**FNfr 25%**	3.97 ± 0.49	0.51 ± 0.15	48.96 ± 7.64	60.20 ± 7.92
**PNfr 100%**	3.90 ± 0.40	0.65 ± 0.11	29.73 ± 2.91	35.54 ± 4.83
**PNfr 50%**	3.86 ± 1.15	0.66 ± 0.10	38.59 ± 12.02	39.87 ± 5.61
**PNfr 25%**	4.65 ± 0.71	0.61 ± 0.05	41.13 ± 9.27	61.55 ± 2.98
**PNfe 100%**	4.46 ± 1.14	0.59 ± 0.12	41.18 ± 7.11	40.52 ± 4.03
**PNfe 50%**	4.78 ± 0.40	0.70 ± 0.11	45.72 ± 9.98	42.30 ± 3.74
**PNfe 25%**	4.87 ± 1.08	0.50 ± 0.10	43.01 ± 5.48	55.21 ± 4.91

FNfe, Fetească neagră fermented grape pomace extract; FNfr, Fetească neagră fresh grape pomace extract; PNfe, Pinot noir fermented grape pomace extract; PNfr, Pinot noir fresh grape pomace extract; MDA, malondialdehyde; SH, total thiol; NOx, total serum nitrates and nitrates; 3NT, 3-nitrotyrosine.

In the plasma there are two major groups of thiols: protein thiols, mainly albumin thiols, and nonprotein thiols or small molecules thiols, such as cysteine (Cys), cysteinylglycine, glutathione, homocysteine and *γ*-glutamylcysteine. Under oxidative stress conditions Cys residues are oxidized resulting in mixed disulphides between protein thiol groups and small molecules thiols, preventing protein thiol oxidation (Yang and Guan, 2015). These disulphide bonds are reversible, creating a dynamic thiol–disulphide homeostasis which is important in antioxidant protection. The dynamic thiol–disulphide dysbalance is implicated in the pathophysiology of many diseases, including cancer (Emre et al., 2017). Turpentine-induced oxidative stress significantly reduced SH (p < 0.001). The pretreatment with GP extracts increased SH (p < 0.001) in a concentration-dependent way. The fresh GP extracts were more efficient, with no significant differences between the two *Vitis vinifera* L. varieties (**Table 3**).

In inflammation NF-kB is a pleiotropic transcription factor that regulates the expression of genes like those for chemokines, cytokines, cell adhesion molecules, growth factors, antioxidant enzymes, iNOS, and others (Silva et al., 2018). NO produced under these conditions is an effector molecule that may have beneficial or harmful effects. At nontoxic concentrations, NO is an effective antioxidant *in vitro* and *in vivo*. If the synthesis is excessive, NO reacts with O_2_^−^ producing high quantities of peroxynitrite (ONOO−), a strong oxidant, which can induce oxidative stress, nitrosative stress, and nitration stress (Lacza et al., 2009). The toxicity hypothesis indicates that high levels of NO induce mitochondrial respiratory inhibition, ATP depletion, DNA deamination, oxidation, and nitration. The hypothesis of the cytoprotective role of NO states that NO protects cells against lipid peroxidation by reaction with sulfhydryl groups in proteins (Jiang et al., 2018). Because flavonols inhibit NO production (Semwal et al., 2016) and NO accumulation (Dai et al., 2013), the reduction of NO may be correlated with the polyphenol content. It was reported that polyphenols can suppress NF-kB activation and translocation into the nucleus of the activated B cells (Annunziata et al., 2020). Plant extracts with anti-inflammatory effects mediated by iNOS inhibition and nitro-oxidative stress reduction may be an adjuvant alternative therapeutic option for tumor cells proliferation and metastasis inhibition (Jiang et al., 2018). Turpentine administration significantly increased NOx (p < 0.001), and pretreatment with GP extracts caused a concentration-dependent reduction of NOx, the fresh GP extracts having a stronger inhibitory effect than the fermented GP extracts. These results correlated with the DPPH test. There were no significant differences between the two *Vitis vinifera* L. varieties in the case of fresh GP extracts (**Table 3**).

3NT is a product of tyrosine nitration mediated by RNS, and it is a marker of inflammation, NO production, nitrative stress, and oxidative stress induced cellular damage (Knight et al., 2018). Induction of inflammation increased 3NT significantly (p < 0.001), and pretreatment with GP extracts reduced 3NT in a concentration-dependent way. There were no important differences between the *Vitis vifera* L. varieties or fresh and fermented GP samples. 3NT correlated with TOS and OSI (**Table 3**).

Because a vicious circle between inflammation, oxidative stress, and ROS formation develops, (Park et al., 2015), the antioxidant therapy involves consecutive anti-inflammatory effects and the analysis of the antioxidant activity indirectly analyzes the anti-inflammatory effects (Shahid et al., 2016). That is the reason why the antioxydant activity of the tested GP extracts can be also considered an anti-inflamatory activity.

### Antiproliferative Effects

Previous data on the effect of GP extracts on cancer cells are limited and the mechanisms are not fully understood. Studies performed on colon cancer cells proved that GP extracts rich in polyphenols had cytotoxic and antiproliferative effects (De Sales et al., 2018; Pérez-Ortiz et al., 2019). Their activity depends on the concentration, target molecule, and environmental conditions. GP extracts have cytoprotective effects toward normal cells and cytotoxic effects toward cancerous cells (Brglez Mojzer et al., 2016). Many data show that polyphenols have anticancer effects due to their antioxidant and anti-inflammatory effects. Their ROS scavenging effects decrease cell proliferation and DNA oxidative damage (Lizarraga et al., 2011). Through the prooxidative effect polyphenols may induce apoptosis of the cancer cells. By inhibiting angiogenesis, polyphenols reduce tumor growth, and by reducing the adhesiveness and invasiveness of cancer cells, reduce the metastatic potential (Brglez Mojzer et al., 2016). Moreover, polyphenols such as resveratrol, quercetin, catechin, and curcumin, proved to influence mitochondrial function. Because cancer cells are high ATP consumers in order to support accelerated proliferation and associated processes, mitochondrial energy metabolism seems to be a proper target in order to cause dysfunction in cancer cells (De Sales et al., 2018). A study performed with Pinot noir GP extract from Brazil, rich in polyphenols with high antioxidant activity, on human hepatocarcinoma HepG2 cells showed that a short-term incubation increased mitochondrial respiration and antioxidant capacity and lowered glycolytic metabolism, and a long-term incubation was cytotoxic and cells died by necrosis (Brglez Mojzer et al., 2016). There are pieces of evidence that the antiproliferative activity of the polyphenol involves also epigenetic mechanisms, such as DNA methylation, histone changes, and micronucleotic acids (miRNAs) that modulate gene expression in cancer (Arora et al., 2019). Therefore, the antiproliferative activity of our fresh and fermented GP extracts with a rich polyphenol content and antioxidant activity was tested on cancer cell lines A549, MDA-MB-231, B164A5, and normal cells HaCat using the Alamar blue viability test.

**Figure 6:**
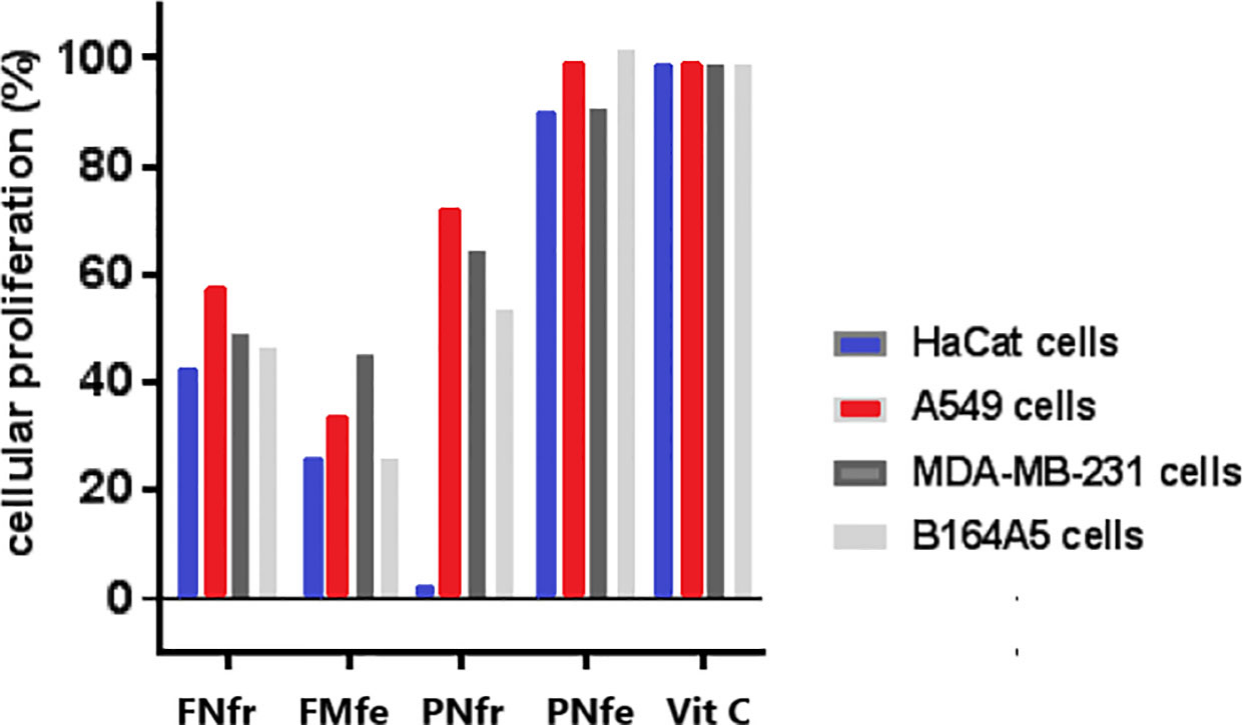
Alamar blue cell proliferation assay. HaCat, keratinocyte; A549, human lung carcinoma; MDA-MB-231, human breast adenocarcinoma; B164A5, murine melanoma; FNfe, Fetească neagră fermented grape pomace extract; FNfr, Fetească neagră fresh grape pomace extract; PNfe, Pinot noir fermented grape pomace extract; PNfr, Pinot noir fresh grape pomace extract.

Proliferation of all four cell lines treated with FNfr was significantly reduced: HaCat (42.58%), A549 (57.27%), MDA-MB-231 (48.89%), and B164A5 (46.59%). FNfe greatly reduced cell proliferation of all cell lines too: HaCat (26.17%), A549 (33.32%), MDA-MB-2 (41%), and B164A5 (29.95%). The antiproliferative effects of FNfe were significantly higher than those of FNfr. PNfr inhibited all cell line proliferation: HaCat (2.39%), A549 (72.36%), MDA-MB-231 (64.47%), and B164A5 (53.75%). Normal cells were found to be more sensitive to stimulation with PNfr. The smallest reductions in cell proliferation were observed for all four cell types after exposure to PNfe: HaCat (89.74%), A549 (98.82%), MDA-MB-231 (90.32%), and B164A5 (101.23%). The antiproliferative effects of PNfr proved to be much stronger than those of PNfe (**Figure 6**). These results were correlated with the polyphenols identified in the GP extracts, respectively FNfe had the highest polyphenol content and the strongest antiproliferative effect. Because the antioxidant activity of the GP extracts was better in the fresh samples, we hypothesized that the prooxidant properties of the GP fermented extracts were involved in the better anti-proliferative effect.

## Conclusions

The phytochemical analysis revealed rich polyphenol content in the *Vitis vinifera* L. var. Fetească neagră and var. Pinot noir pomace extracts, the fermented GP samples having the higher polyphenol concentration. The *Vitis vinifera* L. var. Fetească neagră and var. Pinot noir pomace extracts have antioxidant and anti-inflammatory effects, and the *in vitro* and *in vivo* antioxidant activity were better in the fresh pomace extracts. *In vivo* NO reduction seems to be the cause of a stronger antioxidant effect for the fresh GP extracts. *Vitis vinifera* L. var. Fetească neagră and var. Pinot noir pomace extracts have antiproliferative effects on tested cancer cells and normal cells, and these effects correlate with the higher polyphenol content in the fermented pomace samples.

In conclusion, due to the antioxidant, anti-inflammatory and antiproliferative effects of the *Vitis vinifera* L. var. Fetească neagră and var. Pinot noir pomace extracts, these products can be considered potential agents for nutraceutical formulation in cancer prevention and treatment. Due to the higher polyphenol content the fermented GP extract might be better nutraceuticals.

## Data Availability Statement

The raw data supporting the conclusions of this article will be made available by the authors, without undue reservation, to any qualified researcher.

## Ethics Statement

The animal study was reviewed and approved by the Ethics Committee of the Faculty of Veterinary Medicine, University of Agricultural Sciences and Veterinary Medicine from Cluj-Napoca.

## Author Contributions

SB, AP, MP, LV, CD, and TP conceived and designed the structure of the manuscript and data collection. SB, AP, MP, LV, and TP drafted and revised the manuscript. AP, MP, and LV critically reviewed the manuscript. All authors contributed to the article and approved the submitted version.

## Funding

The publication was also supported by funds from the National Research Development Project to finance excellence (PFE)-37/2018-2020 granted by the Romanian Ministery of Research and Innovation.

## Conflict of Interest

The authors declare that the research was conducted in the absence of any commercial or financial relationships that could be construed as a potential conflict of interest.

The handling editor declared a shared affiliation, though no other collaboration, with several of the authors AP and LV.
